# Presumed endogenous fungal endophthalmitis in a patient with lymphopenia and confirmed SARS-CoV-2 infection

**DOI:** 10.5935/0004-2749.2022-0203

**Published:** 2023-04-10

**Authors:** Erika Moreira Carvalho, Nathalia Silva Santos, Alexandre de Carvalho Mendes Paiva, Fernando Henrique Flores Teixeira, Ana Luiza Biancardi, André Luiz Land Curi

**Affiliations:** 1 Clinical Research Laboratory of Infectious Diseases in Ophthalmology, Fundação Oswaldo Cruz, Rio de Janeiro, RJ, Brazil

Dear Editor,

Since 2019, severe acute respiratory syndrome coronavirus 2 (SARS-CoV-2), a novel coronavirus, has spread worldwide, causing a pandemic. Coronavirus disease 2019 (COVID-19) mainly presents with pulmonary symptoms, but other organs can be affected. If SARS-CoV-2 infects the eye, conjunctivitis and other ocular signs and symptoms such as vascular abnormalities of the retina occur^(1,2)^.

Herein, we report a case of an otherwise healthy 57-year-old woman with pneumonia associated with COVID-19 confirmed by a positive nasopharyngeal swab polymerase chain reaction for SARS-CoV-2. She was hospitalized for 10 days and treated with systemic corticosteroids and oxygen therapy. Blood samples revealed lymphopenia, characterized by a lymphocyte count of 7%-13% (847-1640/mm^3^). No ocular signs or symptoms were observed. However, left eye (LE) visual loss occurred 15 days after hospital discharge.

**Figure 1:**
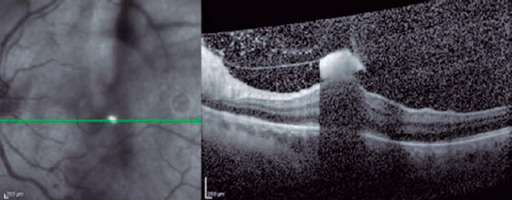
Round-shaped hyperreflective lesion in the inner retinal layers with a shadow effect.

The best-corrected visual acuity (BCVA) was 20/20 in the right eye (RE) and only light perception in the LE. Biomicroscopy revealed anterior vitreous cells in the LE. Fundoscopy showed small, fluffy, creamy white lesions in the posterior pole in the LE. Spectral-domain optical coherence tomography (SD-OCT) images showed vitreous cells, hyaloid thickness with hyperreflective aggregates, and a round-shaped hyperreflective lesion in the inner retinal layers with a shadow effect in the macular area (Figure 1). The ophthalmic examination of the RE was unremarkable.

**Figure 2:**
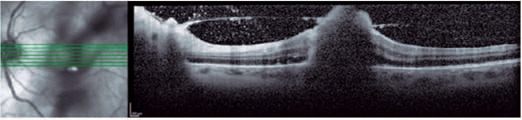
Progression of the hyperreflective lesion with vitreous cells.

The clinical and OCT findings were compatible with endogenous fungal endophthalmitis, and the patient was treated with fluconazole 900 mg orally per day. Despite systemic treatment, the lesions progressed (Figure 2), and the patient underwent pars plana vitrectomy (PPV), which resulted in the total resolution of the inflammation. Direct examination and vitreous culture were negative.

Her final BCVA was 20/20 in the RE and counting fingers in the LE. Two months postoperatively, SD-OCT images showed a hyperreflective area caused by the thickening of the inner retinal layers and anatomical distortion of the macula. The poor visual prognosis was related to macular involvement.

Endogenous endophthalmitis (EE) is a rare ocular inflammation caused by the hematogenous spread of a distant-site infection that infiltrates the internal ocular space through the blood-ocular barrier. It is frequently associated with underlying risk factors such as hospitalization, diabetes mellitus, urinary tract infection, immunosuppression, intravenous drug abuse, and indwelling catheters^(3)^.

Fungi are the main cause of infection among patients with immunosuppression, and neutrophils form the first line of defense against them. Unlike other viral infections, patients with COVID-19 also experience neutrophilia and lymphopenia, which can further increase the risk of opportunistic fungal infections. A high incidence of pulmonary aspergillosis among patients with critical COVID-19 has been reported worldwide^(4)^. In the first examination, our patient already had vitreous involvement. After systemic recovery, even with a normal lymphocyte count and the use of systemic antifungal drugs, the infection progressed, and PPV was performed. Despite a negative vitreous culture and ineffective response to oral fluconazole, OCT images strongly suggested fungal infection. Moreover, the use of fluconazole may have resulted in a negative vitreous culture.

Lymphocytes play a crucial role in the normal immune homeostasis and inflammatory response. Moreover, lymphopenia was reported as an effective and reliable marker of the severity of COVID-19 and hospitalization^(5)^. In the present case, transitory lymphopenia caused by SARS-CoV-2 infection was considered the main risk factor for EE. Therefore, patients with COVID-19 reporting visual symptoms should be carefully evaluated for correct diagnosis and management.
